# The value and use of social media as communication tool in the plant sciences

**DOI:** 10.1186/1746-4811-9-26

**Published:** 2013-07-11

**Authors:** Anne Osterrieder

**Affiliations:** 1 Department of Biological and Medical Sciences, Faculty of Health and Life Sciences, Oxford Brookes University, Gipsy Lane, Headington, Oxford OX3 0BP, UK

**Keywords:** Social media, Social networking, Hashtag, Blogging, Science communication

## Abstract

Social media now complements many parts of our lives. Facebook, Twitter, YouTube and many other social networking sites allow users to share and interact with online content and to connect with like-minded people. Its strengths – rapid dissemination and amplification of content and the ability to lead informal conversations – make it a powerful tool to use in a professional context. This commentary explains the overall concept of social media and offers suggestions on usage and possible types of scientific content. It advises researchers on the potential benefits and how to take a strategic approach towards building a social media presence. It also presents examples of effective social media use within the plant science community. Common reasons for scientists to not engage with social media include the fear of appearing unprofessional, posting something wrong or being misunderstood, or a lack of confidence in their computer skills. With the rapid changes in academic publishing, dissemination and science communication, as well as the rise of ‘altmetrics’ to track online engagement with scientific content, digital literacy will become an essential skill in a scientist’s tool kit.

## Background

Over the last years social media quickly became integrated into many aspects of our daily lives. Websites like Facebook, Twitter or YouTube make it easy to keep in touch with family and friends, to join online conversations and to have easy access to more funny animal videos than there are hours in the day to watch them all. As such, social media still carries the stigma of a frivolous time wasting activity and many scientists are reluctant to engage with it due to lack of time and not seeing the benefits of using it in a professional context [1]. Other barriers include concerns around copyright and legal issues, different research discipline cultures or personal barriers [2]. Yet social media is a powerful professional tool for scientists when used appropriately and efficiently [3,4]. This commentary explains the principles of social media, suggests different ways of using social media platforms in a scientific context and highlights examples of social media use in plant science. It also offers advice on good practice and potential pitfalls regarding sensitive issues around privacy, unpublished data or intellectual property.

## What is social media?

The core principle of social media is the ability to share content with others. In order to upload content, users usually register and create a profile. Depending on the platform and purpose, users are free to choose whether their whole profile or selected content will be publicly accessible or only visible to selected audiences. Types of content might be short status updates, longer text pieces, links, images, audio or video files, publications or CV-related items (Table 1). Other users can then subscribe to a profile to receive regular updates about new content. This connection might automatically be mutual or allow selectiveness depending on the platform. For example, becoming friends in Facebook or connecting in LinkedIn means that both users will see each other’s updates, whereas on Twitter or Google + users can decide whether they want to follow a new contact back.

**Table 1:** The do’s and don’ts of social media

**Content**	**Suggestions**	**Potential pitfalls**	**URLs**
**Short status updates**	Post informative, interesting or engaging updates, e.g. “I am presenting a poster at conference X, come and say hi!”	Avoid boring or too personal updates (“I just had a sandwich”), gossip, personal attacks or excessive negative feelings. Be aware of the sensitive nature of posting about unpublished data, proposals, reviews, collaborators, students etc.	http://www.twitter.com
	“Has anyone got experience with technique Y?”		http://www.facebook.com
**Longer text**	Informative: current research, new papers, conference reports.	Shorter texts (500–700) words are more likely to be read in full. Use images, hyperlinks or multimedia to make text more engaging. Avoid jargon.	http://www.wordpress.com
	Discussion: Opinion pieces, reflections,		http://www.blogger.com
			http://www.tumblr.com
	Creative writing.		https://plus.google.com/
**Photos**	Snapshots from live research, lab/field trips. Data that might not be published otherwise. Use tags or hashtags to contribute to existing image pools and make images accessible.	Avoid using pictures protected under copyright or without appropriate creator attribution, photos of people without having their permission, images you might want to use in a publication.	http://www.flickr.com
			http://www.pinterest.com
			http://www.instagram.com
			http://www.facebook.com
**Video**	Short clips taken with camera or smartphone.	Make use of captions to provide additional information. Think about appropriate length (shorter might reach more people). Avoid using copyrighted music.	http://www.youtube.com
	Interviews, techniques, lectures and talks.		http://www.vimeo.com
			http://www.vine.co
	Data that might not be published otherwise.		
	Creative videos (e.g. songs, cartoons).		
**Links**	Use link shorteners to save space and track clicks.	Avoid posting links without any or with a vague description.	http://www.twitter.com
			http://www.reddit.com
			https://plus.google.com/
			http://www.facebook.com
**Audio**	Soundbites of field trips, events. Longer audio pieces, e.g. interviews, recordings of talks or podcasts.	For longer pieces pay attention to microphone quality and acoustics of the surroundings.	http://www.audioboo.com
	Science songs.		http://www.soundcloud.com
**Publications and CV items**	Invest time to create a professional online presence and keep it up to date.	Before uploading full-text versions or pre-prints, carefully check publisher conditions.	http://www.academia.edu
			http://www.researchgate.net
			http://www.linkedin.com

**Figure 1:**
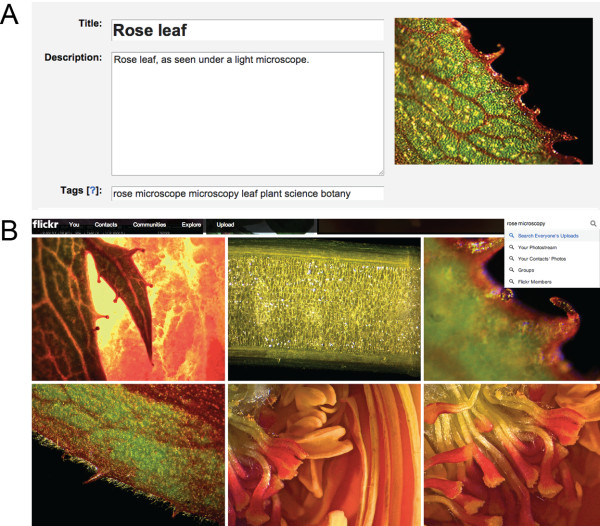
**Using tags to index social media content makes it more accessible.** Example of a rose micrograph uploaded to the photo sharing website Flickr (http://www.flickr.com). **(A)** Besides a meaningful title and image description, tags (= keywords) further extend the accessibility of a photo. In this case, appropriate tags would be ‘rose’, ‘microscope’, ‘microscopy’, ‘leaf’ or ‘plant’. **(B)** The search term ‘rose microscopy’ displays all Flickr photos carrying these specific tags.

Content can be indexed by using tags or hashtags (#). Tags are key words which, when attributed to photos, blog posts etc., allow users to find content relevant to a certain topic more easily. For example, a micrograph of a rose on Flickr might have the tags ‘rose’ and ‘microscopy’ (Figure 1A) and will appear as search result together with all other photos carrying the same tag (Figure 1B). The term ‘hashtag’ describes a similar concept. Adding the hash symbol # in front of any word, phrase or abbreviation turns it into a link that when clicked displays all content containing the particular hashtag in one stream. The concept of a hashtag to initiate and collate conversations about a certain topic is now widely used, both in popular (e.g. for TV shows or radio broadcasts) and academic culture [5]. Examples of academic Twitter hashtags are #phdchat or #ECRchat (early career researcher chat), where researchers network, share writing and career tips and their general joys and woes. The hashtag #highered displays current headlines in Higher Education. Many research areas have their own hashtags, such as #plantpath or #synbio – and if a hashtag does not exist yet, anyone can create one (check out #plantpathsongs). General plant science content and discussions appear under #plantscience or #plantsci and the latter is also used by ‘Sense about Science’ to take questions posed to their plant science panel [6].

## Why use social media?

In the simplest case, social media can provide a highly personalised and relevant ‘Table of Contents’ to keep up to date with current research, popular science and broader issues such as science policy, funding, publishing, or personal career development. Certain social media platforms can also be invaluable tools for professional networking, either within specific subject fields or across different disciplines and professions. Compared to purely academic sites like ResearchGate or Academia.edu, the value of entirely open networks like Twitter or Google + cannot be understated. These open platforms enable dialogue not only between scientists, but also offer opportunities for others to join the conversation: science communicators and journalists, teachers, students, researchers and professionals from other disciplines, as well as other interested non-experts [4].

Actively participating in social media networks allows scientists to disseminate research findings quickly and effectively as well as raise their own profile, of their research groups or institution. More importantly, the interactive nature of the medium can be highly beneficial for scientists by offering new perspectives on their own research through dialogue with peers and non-peers, and helping to establish new collaborations [3]. Communicating science through social media can also help to improve essential scholarly skills such as reflecting, writing for different audiences and developing self-discipline to write regularly [7].

More recently, scientific conferences have started using hashtags to update delegates and support networking during the conference and even before it starts. Covering conferences live on social media allows others to follow and participate in discussions online who might not have been able to attend - or maybe were not even aware of the conference in the first place. This increases dissemination and reach of current research but also requires sensitivity, e.g. in case of unpublished data.

More and more funding agencies, e.g. the European Commission, require open access publications plus outreach activities. The latter normally include scientific publications and press releases. The use of social media in such outreach activities will get an increasingly important measure in milestone planning and reporting in such projects, especially in supranational scientific consortia.

## Where and how to start?

There are many ways to engage with social media. They require different levels of time investment but offer different benefits and rewards. It all starts with the same first step and that is the decision which platform(s) to use and for what purpose. It is worth thinking about the following questions and taking a more strategic approach towards setting up a professional social media presence.

• What do I want to get out of using social media: Keep up to date with current trends and discussions? Expand my professional network? Disseminate my research more widely? Engage non-expert audiences? Who is my audience?

• How much time can or do I want to invest and will the benefits outweigh my time investment? Do I want to use it daily or reserve a block of time regularly to read or create content? Will I need more training?

• How can I incorporate social media most efficiently into my daily workflow? How could I connect accounts or automate things to save time? What other technologies or services can help me to manage my professional online presence?

• How will I be able to evaluate or track the impact of my social media presence? What would my metrics of success be (e.g. subscriber numbers, views, downloads, engagement, comments)?

It is important to note that not only the intended purpose of use, but also the user personality can be a deciding factor when choosing one platform over the other. One site does not fit all and it is worth trying different services before abandoning all of social media completely. For example, some people feel strongly restricted by Twitter’s character limits and prefer Google+, whereas others appreciate Twitter’s brevity. User demographics and tone of discussion can differ significantly between social media sites and this is useful to keep in mind when creating and posting content.

## Consuming, connecting, curating and creating

The easiest, but also most passive way, is to purely consume content. It is entirely acceptable to just read, watch or listen before starting to post, and many people never post anything at all. Usually it is not even necessary to sign up for a social media service to access its content. Registering an account however makes it easier to keep track of content because it opens the possibility to add to favourites or create lists. These features can help with time management, as interesting content can so be marked to return to at a more convenient time. Alternatively, web services like Instapaper, Pocket, Evernote, Feedly, or a simple Word document (ideally stored in a Cloud service) fulfil the same purpose.

Social media users connect with others by following their updates, responding and commenting on them and by favouriting or amplifying content (e.g. by re-tweeting or re-sharing). With the exception of some websites, like Facebook or LinkedIn, it is generally not necessary to know or contact users with public accounts before subscribing to their updates. When using social media for networking, a professional and informative profile is necessary to be found by people with similar interests and to be recognised as an authentic user. The standard photo should be replaced with a personal avatar, which could be a portrait or a representative scientific image. The profile description or short biography should contain relevant keywords such as the subject field, university, location, profession or other interests. Interesting contacts can not only be found via the search function, but also in contact lists of others, as contributors to relevant topics or on curated public subject lists [8,9]. Adding links to social media accounts on presentation slides or posters allows others to connect and keep updated in an informal way. When participating in online discussions it is essential to remember that the internet is a public space where comments are cached, shared and may be spread beyond your control. A good rule of thumb is to only put things online that you would be happy for the rest of the world to see, even if content is seemingly posted to a restricted number of people (screenshots can travel surprisingly far). Respect the social rules of the ‘netiquette, the network etiquette, and be aware of copyright and libel laws (see also advice in Table 1). Most institutions can offer advice or have official guidelines on the use of social media in a professional capacity.

Curating and creating content requires more time and engagement, but can be more rewarding and beneficial in terms of skill and knowledge development and dissemination of plant science to wider audiences. Table 2 shows a small selection of examples, highlighting the wide range of possibilities to use social media for plant science communication. It is important to be realistic about one’s existing skills and the time investment required. Writing a blog entry might take a few hours but will get easier with practice; uploading micrographs or short videos might just be a quick additional step after curating existing data; tweeting an interesting paper takes only a moment. A larger or complex social media project however might benefit from working together with other scientists, professional designers, programmers or communicators.

**Table 2:** Examples of social media use in the plant sciences

	**Example**	**Author/curator**	**URL**
**Plant science news**	‘Functional Biology of Plants’	Martin Hodson and John Bryant	https://www.facebook.com/FunctionalBiologyofPlants
	‘Science and Plants for Schools’	Science and Plants for Schools	https://www.facebook.com/scienceandplants
	‘Kamoun Lab’	Kamoun Lab. The Sainsbury Laboratory	https://twitter.com/kamounlab
	‘Cytogenomics and Epigenetics Laboratory’	Laboratory of Plant Molecular Cytogenetics, University of Sao Paulo	https://www.facebook.com/pages/Cytogenomics-and-Epigenetics-Laboratory/160417117341406
**Plant science blogs**	‘AoB Blog’	Annals of Botany	http://aobblog.com/
	‘Weeding the Gems’	GARNet	http://blog.garnetcommunity.org.uk/
	‘All Under One Leaf’	UK Plant Sciences Federation	http://blog.plantsci.org.uk/
	‘Berry Go Round’	Plant blog carnival	http://berrygoround.wordpress.com/
	‘Plantcellbiology.com’	Anne Osterrieder	http://www.plantcellbiology.com/
	GMO Pundit	David Tribe	http://gmopundit.blogspot.co.uk/
	‘The New York Botanical Garden’	New York Botanical Garden	http://nybg.tumblr.com/
**Magazine-style**	‘Arabidopsis’	GMI Vienna	http://www.scoop.it/t/arabidopsis
	‘Plant Biology Teaching Resources’	Mary Williams	http://www.scoop.it/t/plant-biology-teaching-resouces-higher-education
	‘UBC Botanical Garden’	UBC Botanical Garden and Centre for Plant Research	http://pinterest.com/ubcgarden/
**Video, audio and photos**	‘ChloroFilms’	ChloroFilms	http://www.chlorofilms.org/
	‘Musical Cells’	Anne Osterrieder	http://www.youtube.com/plantendomembrane
	‘Inner Worlds’	John Innes Centre	http://www.youtube.com/user/InnerWorldsJIC
	‘Plant News: audio’	Gatsby Plants	http://www.gatsbyplants.leeds.ac.uk/news_type.php?type=audio
	Plant microscopy	Fernan Federici	http://www.flickr.com/photos/anhedonias/
**Groups**	‘Plant Science’ on Google+		https://plus.google.com/communities/107276823972957651082
	‘Botany’		http://www.reddit.com/r/botany/
	‘UK Plant Sciences Federation	UK Plant Sciences Federation	http://www.linkedin.com/groups/UK-Plant-Sciences-Federation-4498840

## Challenges

The power of social media lies in its interactivity and its strength to amplify the reach of content. At the same time this has the potential to quickly turn into a pitfall. It is therefore essential to be aware of basic rules for using social media. Some of these are included in the rules of ‘netiquette’ and general professional conducts of behaviour. Others depend on the type of content posted and revolve around copyright, intellectual property or confidentiality issues.

With the changes in traditional academic publishing, a major development and yet still a considerable challenge is to identify the impact of scientific content beyond established measures such as citation counts or journal impact factors. ‘Altmetrics’ aim to capture the online activity around a scientific publication by tracking metrics such as downloads, number of readers or amplification and discussion in social networks [10,11]. Researchers can assess their online impact by using professional applications such as Altmetric Explorer [12], Impact Story [13] or a range of other tools [14]. A quick way to get a feel for the online reach of a scientific publication is to pay attention to the article’s altmetrics which are increasingly displayed on journal websites, or to track the reach of a link or hashtag using special social search engines like Topsy [15].

## Conclusion

Even though specific platforms will change in the future, the concept of social media is likely to stay. As such it will become more and more important to engage with social media and become ‘digitally literate’ rather than avoiding or resisting its use at all. There are still many grey areas surrounding social media in society. Unexpected new uses of the medium emerge constantly and carry various opportunities and challenges with them, one good example being citizen live-reporting during recent disasters. While we are starting to establish rules of good practice for some scenarios, society is still trying to evaluate the full impact of others. Understanding social media, and having the knowledge and confidence to use it appropriately and effectively for professional purposes will become essential skills to be included in a scientist’s skills tool kit.

## Competing interests

The author declares that they have no competing interests.

## Authors’ information

AO (@AnneOsterrieder) holds a Research and Science Communication Fellowship at Oxford Brookes University, which allows her to combine her research in plant cell biology with her passion for facilitating science communication and public engagement.
